# Multilocus sequence analysis reveals genetic diversity in *Staphylococcus aureus* isolate of goat with mastitis persistent after treatment with enrofloxacin

**DOI:** 10.1038/s41598-021-96764-z

**Published:** 2021-08-26

**Authors:** Richard Costa Polveiro, Manuela Maria Cavalcante Granja, Thais Coimbra Borba Roldão, Ilderlane Da Silva Lopes, Pedro Marcus Pereira Vidigal, Magna Coroa Lima, Maria Aparecida Scatamburlo Moreira

**Affiliations:** 1 Laboratory of Bacterial Diseases, Sector of Preventive Veterinary Medicine and Public Health, Veterinary Department, Universidade Federal de Viçosa, Viçosa, Minas Gerais 336570-900 Brazil; 2 Núcleo de Análise de Biomoléculas (NuBioMol), Center of Biological Sciences, Universidade Federal de Viçosa, Viçosa, Minas Gerais 336570-900 Brazil

**Keywords:** Microbiology, Bacteria, Bacteriology, Haplotypes, Microbial genetics, Mutation, Computational biology and bioinformatics, Genome informatics, Diseases, Infectious diseases

## Abstract

*Staphylococcus aureus* is one of the main bacterial agents responsible for cases of mastitis in ruminants, playing an important role in the persistence and chronicity of diseases treated with antimicrobials. Using the multilocus sequence typing technique, network approaches and study of the population diversity of microorganisms, we performed analyzes of *S*. *aureus* (ES-GPM) isolated from goats with persistent mastitis (GPM). The most strains of ES-GPM were categorically different phylogenetically from the others and could be divided into two lineages: one with a majority belonging to ES-GPM and the other to varied strains. These two lineages were separated by 27 nuclear polymorphisms. The 43 strains comprised 22 clonal complexes (CCs), of which the ES-GPM strains were present in CC133, CC5 and a new complex formed by the sequence type 4966. The genetic diversity of some alleles showed be greater diversity and polymorphism than others, such as of the *aroE* and *yqiL* genes less than *glpF* gene. In addition, the sequences ES-GPM to the *arc* gene and *glpF* alleles showed the greatest number of mutations for ES-GPM in relation to non-ES-GPM. Therefore, this study identified genetic polymorphisms characteristic of *S*. *aureus* isolated from milk of goats diagnosed with persistent mastitis after the failed treatment with the antibiotic enrofloxacin. This study may help in the future to identify and discriminate this agent in cases of mastitis, and with that, the most appropriate antibiotic treatment can be performed in advance of the appearance of persistent mastitis caused by the agent, reducing the chances of premature culling and animal suffering.

## Introduction

*Staphylococcus aureus* is a major opportunistic pathogen in humans and one of the most important pathogenic Staphylococcus species in veterinary medicine^1^. It appears on the world stage as a challenge in disease chronicity and persistence in antimicrobial treatments^2,3^. *S*. *aureus* is part of a small group of bacteria that can be named as 'persisters' in some diseases^4^, since administration of antibiotic treatments based on correct protocols allows the survival of a distinct form of bacterial subpopulation in an infection^5^. This bacterial agent is one of the most commonly found pathogens in clinical mastitis in several livestock species^1^, such as goats^6^. However, it can cause subclinical, clinical, persistent, gangrenous and difficult-to-treat intramammary infections^7^.

This type of persistent infection could also be identified in the types of mastitis, but it is often just referred to as chronic^8^, and not as an antimicrobial persistence, which can results in the cure failure due to the performance of only the exchange of the antimicrobial, and consequently in premature culling or replacement of incurable animals. In chronic goat’s mastitis cases, the mammary gland can atrophy due to severe damage to the glandular tissue leading to fibrosis and total loss of the ability to produce milk^9^ and can result in the disposal of the animal.

Persistent infections are typically multifactorial and may have mechanisms for different classes of antibiotics, such as β-lactams and fluoroquinolones^10^, and mainly occurs due to the selection of different pathogens capable of evading the immune system^5^, which are genetically prepared to activate the series of stress responses for long-term survival^4^. Persistent mastitis in ruminants^11,12^ raises concerns because it renders ineffective treatments with antibiotics such as enrofloxacin^12^. Enrofloxacin is a fluoroquinolone exclusively developed for use in veterinary medicine^13^ and presents itself as an appropriate choice in some cases of mastitis in goats^14^, being active against major pathogenic bacteria (both Gram-positive and Gram-negative)^15^. Therefore, despite the best possible antimicrobial treatment, failures of bacteriological cure can often occur in mastitis treatments, especially for *S*. *aureus* mastitis, and antimicrobial resistance is considered one of the reasons for low cure rates^16^.

Consequently, identifying the nuclear polymorphisms of *S*. *aureus* to characterize it as persistent during mastitis may be a solution in provide the barcode that can be used to track and identify strains with the same or similar polymorphisms, and thus increase the chances of providing a treatment appropriate antimicrobial. Thus, multi-locus sequence typing (MLST), which use phylogenetic procedures on the nucleotide sequences of allelic locus used in MLST are being widely used for identification and determination of phylogenetic relationships between isolates^17^. On the other hand, the haplotype network presents itself as an alternative representation of the clonal genealogy of bacterial isolates^18^ and complementary to the phylogeography of *S*. *aureus*. In addition, reconstructing bacterial haplotypes is required when choosing the right treatments for diseases caused by specific haplotypes in a population^19^.

Population analyses of bacteria based on data from different loci using MLST schemes can provide important epidemiological results, especially if confronted with different methods involving genealogical typing^20^. Therefore, it was the aim of this study is to investigate the genetic diversity of *S*. *aureus* (ES-GPM) isolated from goats with persistent mastitis (GPM) through MLST scheme and compare with other sequence of *S*. *aureus* isolated from different other types of mastitis (non-ES-GPM) and hosts that are deposited in the PubMLST database^21^, using phylogeographical, phylogenetic, and diversity approaches.

## Results

**Figure 1:**
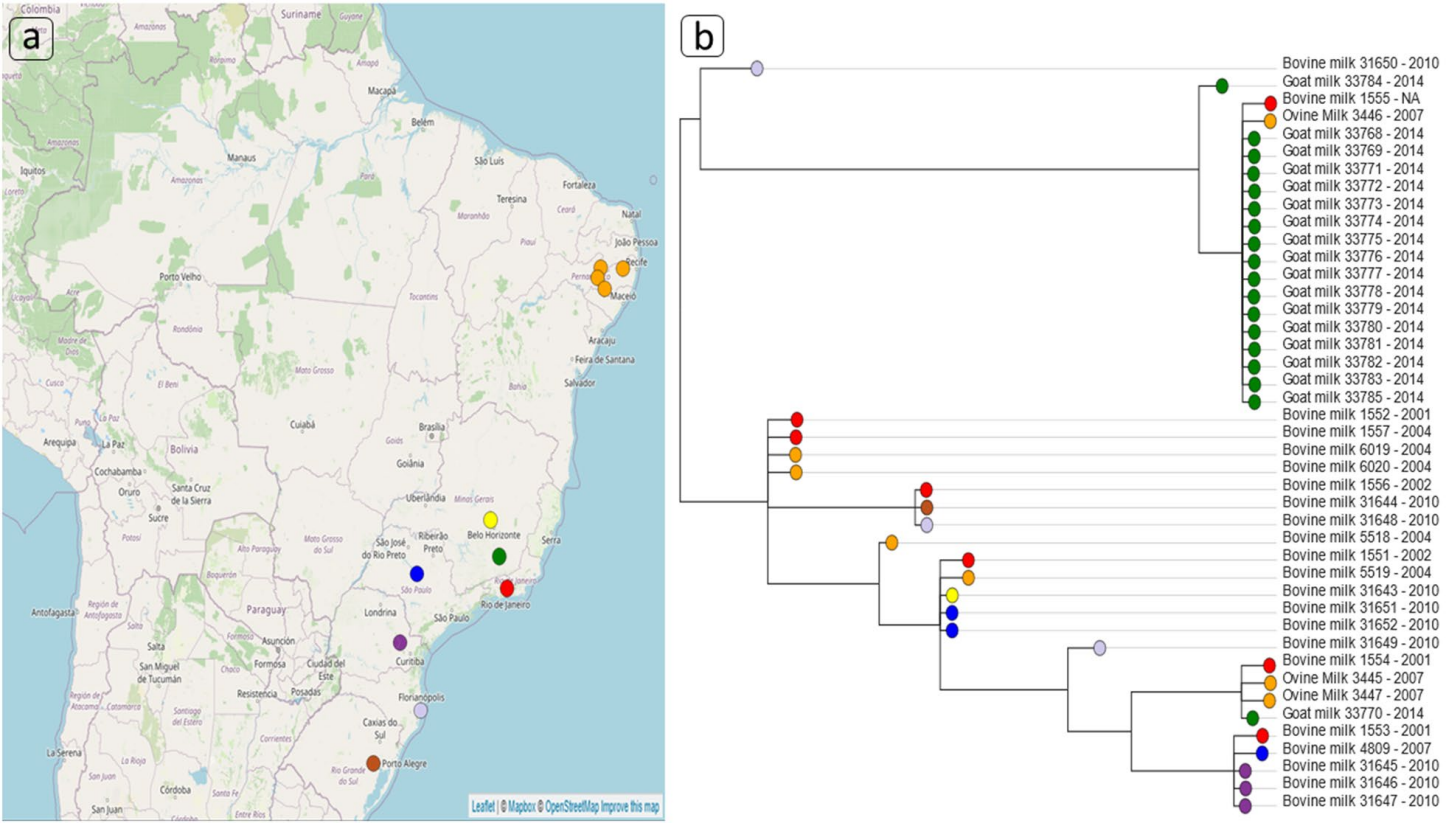
Geographic distribution, geospatial information, and phylogenetic analysis of isolates of *Staphylococcus aureus* in milk from Brazilian herds. (**a**) Geographic distribution of the 43 isolates among the seven Brazilian states. Each dot on the map represents the origin location of mastitic milk: Orange—Pernambuco (Northeast / BR), yellow—Minas Gerais (Southeast / BR), and green (ES-GPM)—Minas Gerais (Southeast / BR)—Minas Gerais (Southeast / BR), red—Rio de Janeiro (Southeast / BR), blue—São Paulo (Southeast / BR), purple—Paraná (South / BR), gray—Santa Catarina (South / BR), brown—Rio Grande do Sul (South / BR). Created with Microreact version 5.93.0 (at http://microreact.org). (**b**) Phylogenetic tree structured with metadata in Microreact, with the colors referenced to each Brazilian state analyzed. The tip labels were detailed with the source of the samples, identification of the isolates (ID) and lastly this is the year in which it was collected and analyzed. The tree was visualized using Figtree software and the fully interactive version of our phylogenetic tree and including geospatial information can be found at https://microreact.org/project/puA-MDz8o. Created with Microreact version 5.93.0 (at ) and by Figtree software version 1.4.4.

A total of 43 isolates were analyzed in this study. Within these 43, 18 (ES-GPM) isolates from the milk of animals were diagnosed with GPM at Capril UFV, named Minas Gerai—LDBAC (Table S1). In addition, another 25 isolates of animals diagnosed with mastitis were selected from the PubMLST database (https://pubmlst.org/organisms/staphylococcus-aureus/); these are shown in supplementary Table S3. As a result of this search of the database, 22 bovine mastitis and three ovine isolates were referenced. The total sequences obtained originated from seven different Brazilian states: Rio de Janeiro (n = 7), Pernambuco (n = 7), São Paulo (n = 7), Minas Gerais (n = 1), Rio Grande do Sul (n = 1), Paraná (n = 3), Santa Catarina (n = 3) and Minas Gerais-LDBAC (n = 18) regarding the ES-GPM sequences (Fig. 1a and b). These analyzed sequences represent all the sequences deposited at (https://pubmlst.org/organisms/staphylococcus-aureus/) referring to animal mastitis in Brazil up to 09/28/2019. The MICs ranged from 0.125 to 16 μg/mL in the isolates prior to treatment, whereas in the isolates after treatment with enrofloxacin antibiotic, the antimicrobial MICs ranged from 0.19 to 16 μg/mL, with only samples of IDs 33784 and 33785 showing 16 μg/mL, as previously reported^22^.

### Phylogeography, STs and CCs

In Fig. 1, the phylogeographic distribution of *S*. *aureus* isolates are characterized, relating the phylogeny of the strains to the isolation sites of the sample, which showed little association between the origin of the isolate and the phylogenetic structure found. Despite the apparent small number of sequences as shown in Fig. 1, these MLST data represent the totality of Brazilian sequences deposited from *S*. *aureus* that infected dairy animals. However, strains related to ovine and caprine mastitis apparently had isolates from bovine mastitis as common ancestors. In addition, ES-GPM-related strains were highly associated in clades or clusters, which may be because few different strains causing GPM to circulate among animals within the same goat herd.

On the other hand, strains related to bovine mastitis demonstrated phylogenetic ramifications along the branches of the phylogenetic tree (Fig. 1), regardless of the Brazilian region studied, which implies greater nucleotide polymorphism. Strains related to ES-GPM are apparently little related phylogenetically to the other two host species (ovine and bovines) and show no clear phylogenetic distinction, as can be seen by strains from bovine ID 1555 and ovine ID 3446 (Fig. 1). However, even though most of the ES-GPM-related strains remained close phylogenetically, the strain ID 33770 (ST 5; Fig. 1) distanced itself from the other ES-GPM strains, becoming close to those from ovine and bovines. In addition, IDS strains 33784 and 33770 were harvested prior to treatment with enrofloxacin as listed in Table S1, and this possibly determined that they were phylogenetically more distant from other ES-GPM strain and that also presented as a different ST.

**Figure 2:**
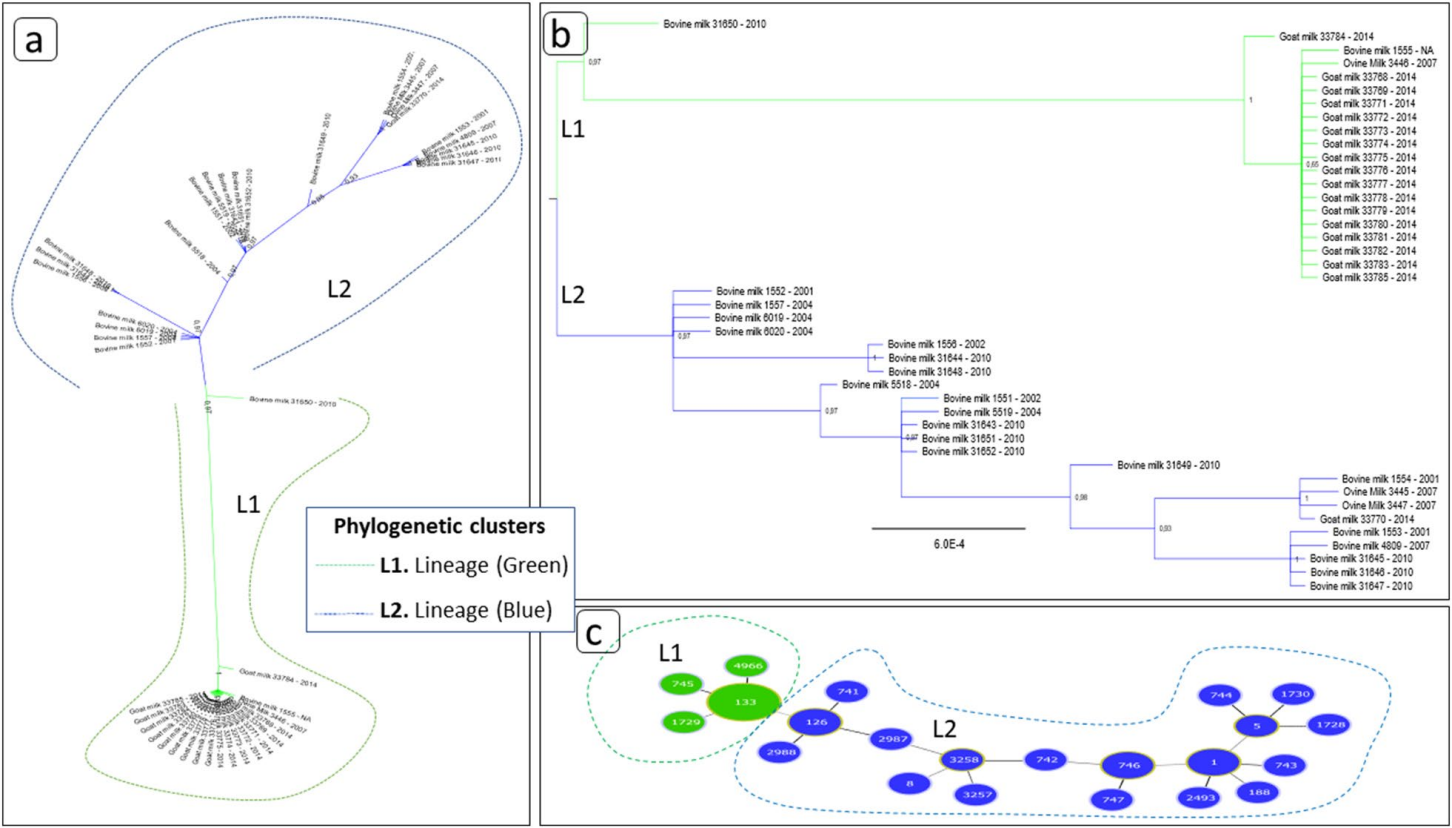
Phylogenetic tree and MS tree. (**a**). Tree and phylogenetic structure in line with Multiloccus Sequence Tree (MLST tree) subdivided phylogenetically into clades (L1 and L2). Created with Figtree software version 1.4.4. | (**b**). Phylogenetic tree represented in a cladogram with more information on the isolates, such as: sample source, sample identification (ID) and year of sample collection. Created with Figtree software version 1.4.4. | (**c**). MS tree. The MS tree of the 22 STs was inferred by Phyloviz on the basis of allelic profiles. Each circle indicated a ST (node), and a larger size of the circle corresponded to a larger number of strains included. The dash lines separated two lineages L1 to L2. Minimum spanning tree reflects the clonal relationships of the sequence types (STs) for 43 *Staphylococcus aureus* bacteria subdivided between two strains (L1/green and L2/blue) constructed using goeBURST characterized as clonal complex (CCs). The background colors indicated different CCs. Created with PHYLOViZ software.

The corresponding phylogenetic analyses (Fig. 2a,b) showed high consistency with the Multilocus Sequence Tree (MS tree) (Fig. 2c), providing better resolution when considering the seven (AT) genes characterized for *S*. *aureus* (Fig. 2a,b) than if a single *housekeeping* gene were used. In addition, these analyses also presented robust topology with high bootstrap values, dividing the studied population into two lineages (Fig. 2a). The phylogenetic tree based on housekeeping genes or concatenated maintenance genes, as shown in Fig. 2b, indicated moderate differentiation among populations, as also shown in Fig. 1b. Nevertheless, the 43 strains were divided into two clusters, called Lineage 1 (L1) and Lineage 2 (L2), being characterized by four CCs and another 18 CCs, respectively.

Accordingly, each lineage cluster (Fig. 2a) represents one or more phylogenetic associations that, concomitantly, also determined the formation of CCs in Fig. 2c, and thus 20 STs with their concatenated sequences formed L1, and 23 formed L2. The high nucleotide identity caused L1 to form a smaller cluster of CCs (Fig. 2) compared to L2 (Fig. 2).

Among the 22 isolates from bovines, 16 different STs were identified, while for the three ovine isolates, three STs were identified. Of the 18 goat isolates from LDBAC-UFV, three STs were also identified, which totaled 22 STs. These isolates were characterized in 22 CCs as shown in Fig. 2c. Other information, such as the AT variation of each of the seven housekeeping that characterize ST, can be found in Table S3.

**Figure 3:**
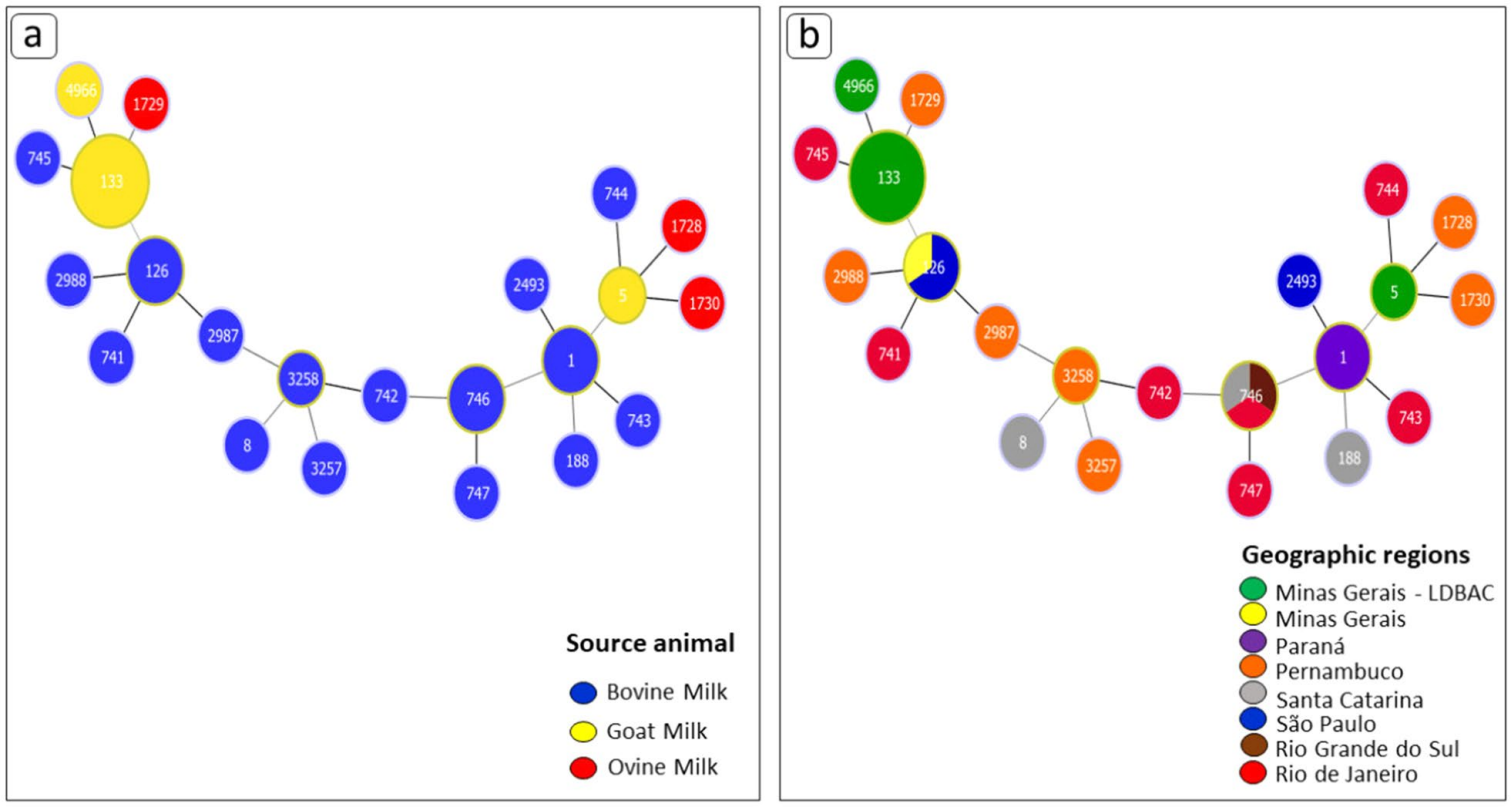
The MS tree of the 22 STs was inferred by Phyloviz based on allelic profiles. Each circle indicated a ST (node) or complex clonal (CC), and a larger size of the circle corresponded to a larger number of strains included, in other words, size of the circle is logarithmically proportional to the number of strains represented by the ST. The background colors indicated different CCs. (**a**) Each color represents a different host for *Staphylococcus aureus* (bovine, caprine and ovine) obtained from milk of animals with mastitis. The circles with blue colors denote the source of the sample from bovine, yellow from goat and red from ovine. Created with PHYLOViZ software. (**b**) The isolates are colored according to isolated areas, distinguishing between samples from 7 Brazilian states. The CC referenced in green are from milk of goats with persistent goat mastitis (GPM). Created with PHYLOViZ software.

A larger number of smaller-sized blue CC circles are shown in Fig. 3a, which demonstrates a greater variability between the ATs of *S*. *aureus* STs that cause bovine mastitis. On the other hand, the STs of the same bacteriological agent that affects goats and ovine are at opposite ends of the MS tree, and in some cases with CCs of larger size, showing less variability and clustering of STs by several isolates. Among these, we highlight CC133, CC5 and CC4966 in green in Fig. 3b, which correspond to GPM and ES-GPM. In addition, the fact that the STs of goat and ovine ES-GPM are positioned at two opposite points of the MS tree and connected by the CCs in blue (bovine samples), can demonstrate that the colonization of *S*. *aureus* in ovine and goats, and ES-GPM, are genetic conditions that arose from bovine strains at different times, determining the same pathogenic condition.

In the MS tree in Fig. 3b, the description of the CCs can be observed in relation to the states of origin of the samples. Some states showed the greatest variability in STs and, because of that, larger CCs as well, such as the state of Rio de Janeiro (red) and Pernambuco (orange), which each held seven different STs. The CC746, of IDs 1556, 31644 and 31648 (from bovine samples—Table S3), was the most present in the largest number of states, such as in Santa Catarina, Rio de Janeiro and Rio Grande do Sul, and therefore CC126, of IDs 31643, 31651 and 31652, were present in the states of Minas Gerais and São Paulo.

### Haplotypic network of *S*. *aureus* for source animal and geographical distribution

**Figure 4:**
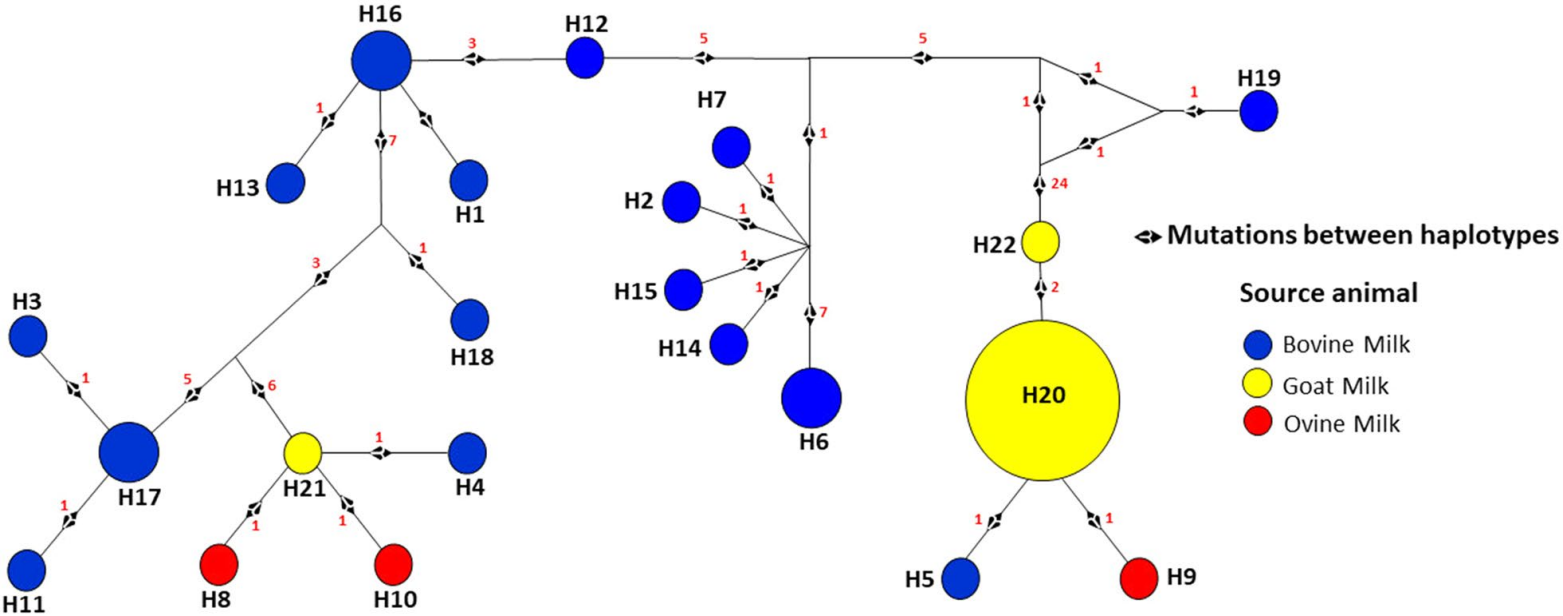
Haplotypical network of multilocus sequence typing alleles *arcC*, *aroE*, *gmk*, *pta*, *tpi*, *glpF* e *yqiL* of *Staphylococcus aureus* obtained from milk of animals with mastitis. Hosts: Yellow—Caprine; Blue—Bovine; and Red—Sheep. Red numbers between haplotypes—polymorphic sites. The colors separating the haplotypes refer to the groups formed in the MS tree. Constructed with Network 4.6.1.0 software.

**Figure 5:**
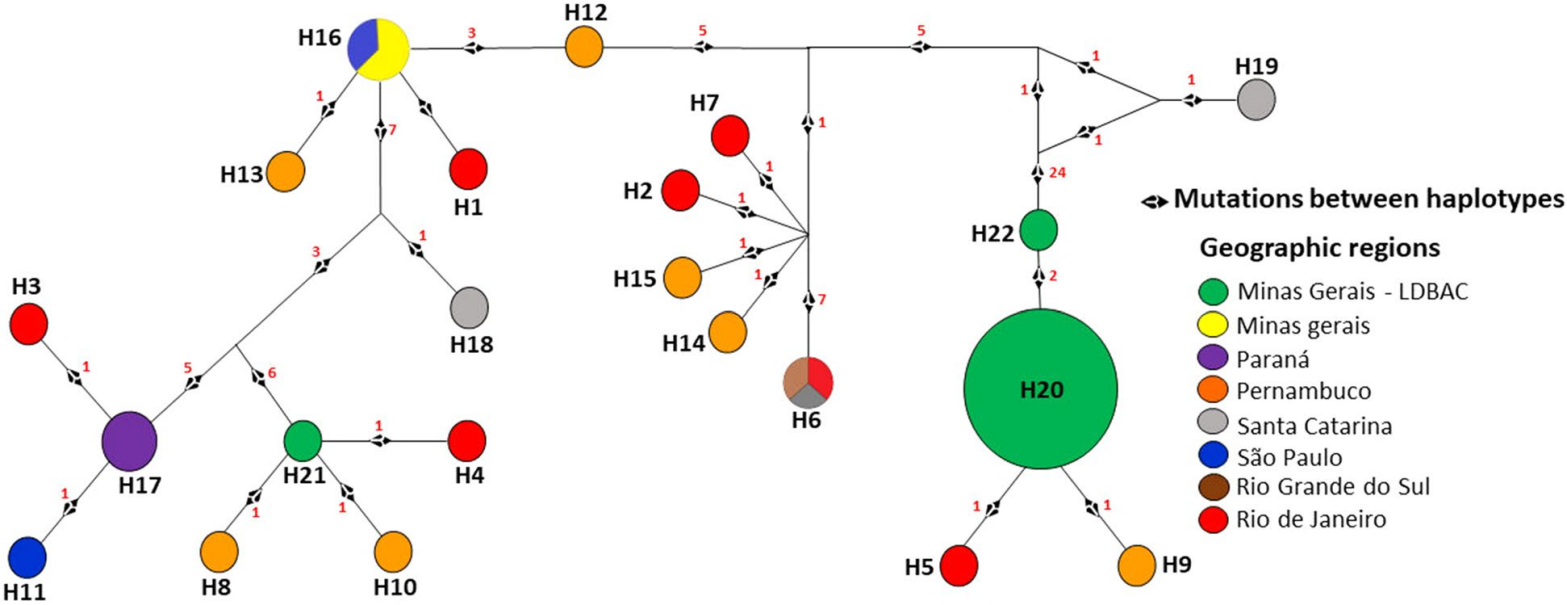
Haplotypical network of multilocus sequence typing alleles *arcC*, *aroE*, *gmk*, *pta*, *tpi*, *glpF* and *yqiL* of *Staphylococcus aureus*, obtained from milk of animals with mastitis and based on the geographic distribution of Brazil. The distribution refers to the states of Minas Gerais—yellow and green for Minas Gerais—LDBAC, Pernambuco—orange, Paraná—purple, Rio de Janeiro—red, Rio Grande do Sul—brown, Santa Catarina—gray and São Paulo—blue. Red numbers between haplotypes—mutations sites. The colors separating the haplotypes refer to the groups formed in the phylogenetic tree and MS tree. Constructed with Network 4.6.1.0 software.

In this analysis, with the concatenation of the seven MLST genes from *S*. *aureus*, the 43 sequences were grouped into 22 haplotypes (Table 1). The H20 haplotype (Figs. 4 and 5), referring to the ES-GPM sequences, was the point in the network that presented the greatest number of aggregated samples, being exclusively of goats, and thus of the ES-GPM from Minas Gerais. The bovines showed a greater number of different haplotypes (72.73%), followed by goats (13.64%) and ovine (13.64%; Fig. 4). The haplotypic diversity (Hd) of the broad population of *S*. *aureus* (seven MLST housekeeping genes) was higher (0.8571; Table 1), represented by the high number of haplotypes formed. The haplotypes referring to the goat and ovine samples show that their ancestors have always belonged to *S. aureus* from bovine isolates (Fig. 4).

**Table 1:** Molecular diversity indices of wide populations by multilocus sequence typing genes of *Staphylococcus aureus*.

	*arcC*	*aroE*	*glpF*	*gmk*	*pta*	*tpi*	*yqiL*	Concatenated
*T*^*a*^	456	456	465	417	474	402	516	3186
*N*^*b*^	43	43	43	43	43	43	43	43
*h*^*c*^	5	8	4	5	6	4	10	22
*Hd*^*d*^	0.6844	0.7375	0.5548	0.6844	0.6534	0.6744	0.7907	0.8571
*S*^*e*^	6	13	5	9	6	7	15	61
S*	4	10	3	7	4	7	12	47

***T***^***a***^: length of DNA sequences (pb); ***N***^b^: number of sequences; ***h***^c^: number of haplotypes; ***Hd***^d^: haplotypic diversity; ***S***^e^: polymorphic sites; S* number of parsimony informational sites.

Interestingly, the distribution patterns of CCs found in the MS tree (Fig. 3) were like those of the haplotype networks (Figs. 4 and 5). Thus, H20, H22, H5 and H9 (Fig. 4) corresponded to CC133, CC4966, CC745, CC1729, respectively, while H21, H8, H10 and H4 corresponded to C5, CC1730, CC1728 and CC744. In addition, C133 (Fig. 3) or H20 (Fig. 4) had between 24 and 27 mutations separating them from the other haplotypes, and the groups corresponding to C5 (Fig. 3) or H21 (Fig. 4) were separated from the rest of the network groups by 6 to 7 mutations. However, most haplotypes (72.73%) were separated by one mutation, while the rest (23.81%) were separated by between 2 and 27 mutations.

The high number of mutations presented from H22 established the separation of the groups, and Line 1 (L1; 22.73%) was formed by haplotypes H5, H9, H20 and H22. With this, H22 was positioned exactly where the two different clusters/strains are separated (Figs. 4 and 5), between H22 and H19, a result corresponding to the analysis of Fig. 2b. On the other hand, the H21, H8, H10 and H4 haplotypes were part of the Line 2 (L2) lineage, with H21 or C5 being the only GPM and ES-GPM haplotypes that did not group with L1.

Of the 22 haplotypes found, only 13% were goats and H20 was the most common (n = 16, 37.2%), formed by ES-GPM collected at UFV, Minas Gerais. The H20 haplotype (Minas Gerais—LDBAC) from goat strains proved to be a common ancestor of the H22 (Minas Gerais—LDBAC), H9 (Pernambuco), and H5 (Rio de Janeiro) haplotypes (Fig. 5). However, the H19 haplotype (Santa Catarina) was the haplotype that differed most in the number of mutations from ancestors and other haplotypes; however, a greater number of samples from this state could help to reduce this isolation and complement the network.

Other haplotypes belong to the lineage L1, H20 and H5 with bovine and ovine sequences, respectively, showed only one mutation point between them. The H19 haplotype, also belonging to this strain, was the most distinct from the others. The L2 line (77.27%) was formed by the other haplotypes, with cattle representing 82.35% of this line (Fig. 4). The number of mutations represented in the haplotypic network of Figs. 4 and 5, suggest that the L1 strain can be highly divergent from the isolates of the L2 strain.

The H6 haplotype, referring to the bovine *S*. *aureus* sequences, was the haplotype with the greatest dispersion among the states, presenting itself in the states of the southern region (Santa Catarina and Rio Grande do Sul) and in the southeast region (Rio de Janeiro). In addition, H16 presented itself as a common ancestor and divisor of the haplotypical network, branching out several haplotypes, such as H13 and H12 (Pernambuco) and H1 (Rio de Janeiro). In the entire haplotype network referring to the different Brazilian regions (Fig. 5), the states of Rio de Janeiro and Pernambuco were the ones that presented the greatest number of haplotype diversities.

### Haplotypic networks of individual genes by hosts

The individual analysis of the seven genes of *S*. *aureus* isolates in the different hosts revealed the formation of 4–10 different haplotypes (Table 1). While the haplotype network analysis of the concatenated housekeeping genes (large population) revealed a greater specificity of the haplotypes for their host, in the analysis of the seven housekeeping genes separately there was predominantly an aggregation of the isolates in a non-host-specific manner (Fig. S1 and S2). The haplotypes referring to milk samples of cow or cattle origin appear to be more specific; at the time, the haplotypes referring to ovine and goats showed greater plurality.

The frequency of different hosts that shared AT of *S*. *aureus* in the same haplotype was low: 9% on average. Only the H4 haplotype for the *yqiL* gene showed 100%, in which it grouped three different hosts in the same proportion. The *yqiL* and *aroE* genes showed the highest values of haplotypic diversity: 0.7907 and 0.7375, respectively, while *glpF* had the lowest value: 0.5548 (Table 1). The greatest genetic variability observed for the *yqiL* and *aroE* genes, to the detriment of the haplotypic diversity values, demonstrated that these genes are more mutable in relation to *glpF*.

The genetic diversity shown in the *arc*, *tpi*, *pta* and *gmk* genes were very similar (Table 1). The arc and *tpi* genes showed a remarkably similar distribution of haplotypes in each host. However, the *pta* and *gmk* genes showed more specific haplotypes for ovines and goats than the other genes shown in supplementary Figure S1 and S2. However, the *pta* gene (Fig. S2) appears to have a greater haplotype specificity for goats and ovines than the other genes.

### Sequence diversity

In order to assess the general sequence diversity of the seven MLST loci of the isolates under study, the average GC (genomic DNA base composition) content, the number of polymorphic sites, the ratio of non-synonymous (dN) to synonymous (dS) substitutions and nucleotide diversity (π) were calculated and are shown in Table 2. Sequence alignment of each MLST locus showed no insertion/deletion. Concatenated sequences for the seven loci were 3186 bp in length with a diversity index π of 0.00670. The mean GC content of the MLST gene fragments ranged from 30.10% for *aroE* to 40.9% for *glpF*. The nucleotide diversity index π ranged from 0.00346 (*glpF*) to 0.01119 (*aroE*; Table 2). The number of polymorphic sites varied from 1.07% for *glpF* to 2.93% for *aroE*, the most polymorphic locus (Table 2). Interestingly, the *glpF* sequence showed the lowest haplotypic diversity of all (Table 1) and the highest average G + C content (Table 2).

**Table 2:** Nucleotide and allelic sequence diversity for whole population of seven housekeeping genes of *Staphylococcus aureus* in multilocus sequence typing.

Locus	Length (bp)	Average G + C content (%)	S/M	No. of polymorphic sites (%)	SNPs	Average *d*N/*d*S ratio (%)	No. of nonsense mutation	Nucleotide diversity (π)
*arc*	456	38.30	6	1.32	2	0.239	1	0.00354
*aroE*	443	30.10	14	2.93	3	0.432	2	0.01119
*glpF*	465	40.90	5	1.07	2	0.228	1	0.00346
*Gmk*	417	33.50	9	2.15	2	0.151	–	0.00762
*Pta*	474	36.20	6	1.26	2	0.383	–	0.00447
*Tpi*	402	37.50	7	1.74	0	0.095	–	0.00766
*yqiL*	516	37.90	15	2.90	3	0.173	2	0.00900
Concatenated	3186	36.40	62	1.94	14	0.261	6	0.00670

Variable sites (**S**)/mutations(**M**); Number of singletons mutations (**SNPs**); Nucleotide diversity, Pi (**π**); All locus contains 43 alleles.

The dN/dS values ranged from 0.095 (*tpi*) to 0.432 (*aroE*; Table 2) indicating that all seven MLST loci exhibit purifying selection, since the dN/dS ratio indicates purifying selection if dN/dS < 1^17^. In general, the greatest number of mutations was observed for the whole population of sequences (Table 2), with emphasis for *aroE* and *yqiL* alleles. In addition, the sequences of ES-GPM (supplementary Table S4) also demonstrated that the *aroE* and *yqiL* alleles were present more polymorphisms. Thereby, the sequences ES-GPM to the *arcC* (66%) and *glpF* (60%) alleles showed the greatest number of mutations for ES-GPM in relation to non-ES-GPM (Table S4).

### Nonsense mutations in *arC, aroE*, *glpF*, *yqiL* and test of recombination

Six nonsense mutations were unexpectedly found in four distinct alleles and, interestingly, only in the lineage L2, being denoted in *arcC* (CAA → TAA) at position 111 of ST 2988 (bovine); *aroE* (CAG → TAG, AGA → TGA) at positions 274 and 57, STs 742 and 3258, respectively (bovine); *glpF* (CAA → TAA) at positions 363 in ST 1728 (ovine); *yqiL* (CAG → TAG, AAA → TAA) at positions 170 in ST 1730 and at positions 495 for the STs 1730 (ovine), 744 (bovine), 1728 (ovine), 1730 (ovine) and 5 (goat). Interestingly, nonsense mutations were not identified in the new ST 4966.

The *phi* test is a rapid statistically efficient test for recombination. The P-value generated from the *phi* test for all 22 STs was *p* = 4.053E−5 (Table 3) and for L2 was *p* = 5.413E−03, which indicates significant incidence of recombination across the whole population and L2. However, for L1 it was not possible to establish the value (NA) by the program used, which suggested that there was no significant evidence of recombination for L1 or, if not, partly since there are very few informative sites. Furthermore, the *phi* test for each AT did not demonstrate a significant result.

**Table 3:** Recombination test and estimation of *Staphylococcus aureus*.

Population(n)	*Phi* test	Recombination analysis					Linkage disequilibrium	
		theta/site	rho/site	rho/theta	LB 95%	UB 95%	IAs	*P* value
All (22 STs)	4.053E−5	4.854E−03	1.05E−03	2.16E−01	4.534E−04	1.698E−03	0.0052	< 1.00E10−04
L1 (4 STs)	NA	6.849E−04	5.65E−01	826.053E+00	7.301E−04	2.777E+00	0.0001	1.98E10−02
L2 (18 STs)	5.413E−03	3.618E−03	2.26E−03	6.24E−01	5.134E+00	11.102 E+00	0.0014	< 1.00E10−04

Contains 22 Sequence types (**All**); Lineage 1 (**L1**); Lineage 2 (**L2**); Standard association of index (**IAs**); Upper bound 95% (**LB)**; Lower bound 95% (**UB).**

The detecting per-site ρ/θ (rho/theta) value for the 22 STs was 2.16E01 (Table 3), suggesting that point mutation was 4.63-fold more likely to occur than recombination at the level of whole population. The values of ρ/θ ratio were 6.24E−01 for lineage L2, which likewise suggests point mutation in this lineage to be 1.62-fold more likely to occur than recombination. However, the ρ/θ ratio for L1 could indicate a high recombination rate, but the *phi* test did not indicate that there would be evidence, as previously reported in the text. The IAs values were 0.0052 (*P* =  < 1.00E10−04) and 0.0014 (*P* =  < 1.00E10−04; Table 3) for all 22 STs and L2, respectively, indicating a tendency toward linkage disequilibrium between the alleles of L2 at the level of whole population (all 22 STs). This result indicates that clonal relationship and recombination were not sufficient to break down the linkage disequilibrium for all 22 STs and L2. However, for L1 a tendency toward free recombination between the alleles in lineage was suggested.

## Discussion

In recent years, persistent infections have shown an important role in the relapse and recalcitrance of infections, moreover they are likely to help spread antibiotic resistance^23^, being persistence a potential critical trigger for therapeutic failures^4^. Overall, phylogenetic analyses resulted in the establishment of two lineages of cases of bovine, ovine and caprine mastitis in different states of Brazil, and highly clonal ES-GPM unresponsive to the antibiotic enrofloxacin, in a single herd of goats. The 43 isolates that formed the 22 CCs provided further evidence that geographic isolation was not the primary factor leading to moderate genetic differentiation of *S*. *aureus* ES-GPM and Non-ES-GPM.

*S*. *aureus* isolates from animals are commonly assigned to host-specific CCs^24^, and CC133 was the main group of characterized isolates belonging to ES-GPM in this study, in addition to the presence of the CC5 complex. In previous studies, CC133 has been associated with small ruminants^25^ and has been specifically assigned to goat, ovine and bovine isolates in several different countries^24^ and in Brazil^26^. According Aires-de-Sousa et al.^27^, the CC133 of *S*. *aureus* may have adapted to small ruminants, with human ancestry, due to the adaptive diversification of the genome resulting from allelic variation, the loss of genes or horizontal acquisition of mobile genetic elements.

The CC5 was also identified as belonging to the ES-GPM association but in a separate lineage from CC133. In addition, CC5 is recognized as a common clonal complex and generalized to several hosts, and is among the most prevalent clones that cause hospital infections in humans and causes methicillin-resistant *S*. *aureus* (MRSA)^28^. CC5 has also been characterized in bovine mastitis^29^, in buffalo milk^30^ and also isolated in foods such as samples of milk and dairy products^31^. Consequently, the persistent *S*. *aureus* lineage, may be silently acting on persistent infections along with CC133 and CC5 in other cases of mastitis or even in other clonal complexes not studied here.

RABELLO et al.^32^ suggests that the prevalence of a limited number of clones is strictly related to mastitis in different herds and HOEKSTRA et al.^33^ demonstrated that the same genotype of *S*. *aureus* can cause clinical and subclinical mastitis in goats and ovine. However, diversity analyses implemented using different techniques indicate that there is a difference between sequences by type of mastitis, such as ES-GPM. This indicates that questions of agent selection or challenge^3^ can trigger the persistence^34^ of mastitis, and with this, questions related to the history of choosing certain classes of antibiotics in treatments and adaptations of the agent must be taken into account in the analysis of MLST in mastitis, as in cases of MRSA or MSSA (methicillin-susceptible *S*. *aureus*). As the mutations that lead bacteria to persist to enrofloxacin and other fluoroquinolones are still being determined^12,35–37^, more complete studies, with total genome sequencing^38^ and biocomputational analyses should be better implemented in the future to elucidate these issues.

As was reported above, we did not find high genetic variability in this study, but nevertheless, it has been reported that cases of animal mastitis by *S*. *aureus* in small regions of Brazil are related to low genetic variability and a small clonal population^27^. Furthermore, a greater number of sequences could better demonstrate the genetic diversity, patterns of distribution and evolutionary history of this agent in dairy animals. On the other hand, in cases with a large number of isolates collected from different regions of Norway, the strains were closely related genetically, and their clonal population was responsible for most cases of mastitis by *S*. *aureus* in domestic ruminants^39^. The low diversity of *S*. *aureus* in milk samples in studies with ruminants^24,27,40^ may be related to its later diversification from *S. aureus* associated with humans through a combination of foreign DNA acquisition and gene decay^41^ and also that their strains, in general, are young in relation to the species^38^. However, in this study the strains related to bovine mastitis demonstrated greater nucleotide polymorphism possibly associated with evolutionary pressures under the pathogen due to factors related to adaptation of a species to optimize the process of infection, escape host immune response, and also as possible adaptation to a different environmental niches and use of antibiotics that impacts on the evolution of certain core genes^42^.

In general, for isolates of *S*. *aureus* from invasive disease the r/m per allele parameter is approximately 1:1^43^, which means that the isolates would have the same probability of diversifying in their large population by recombination as by point mutation. However, in humans, the genetic variability of *S*. *aureus* may be mainly associated with point mutation, since alleles are up to 15 times more likely to change by point mutation than by recombination^44^, similar to the results of our study. Conversely, in *S*. *aureus* adapted to cattle, it was found by MLST alleles that a nucleotide substitution was more likely to be due to recombination than to point mutation, and equally likely in humans^45^. Nevertheless, there is an extensive difference between the *S*. *aureus* genomes associated with cattle and those isolated from humans^41^. These differences between genomes and their r/m ratios demonstrate that there are gaps in the understanding of the diversification behavior of *S*. *aureus* between different hosts. In addition, subtle changes in strain due to single non-synonymous point mutation in *S*. *aureus* may be involved with persistence to antibiotics^46^ as diagnosed in GPMs unresponsive to treatment with enrofloxacin.

Nevertheless, there is evidence of widespread homologous recombination in the core genome of *S*. *aureus* in studies of animal-associated strains of ovines, bovines, and poultry^38^, due to the performance of mobile genetic elements (MGEs), which generate a landscape of hotspots in the core genome^38^. Furthermore, there are concerns about the exchange of *S*. *aureus* CCs between animals and humans^47,48^, in which strains of this agent have been previously described in the literature as being capable of causing disease in animals and humans^49^. Therefore, the probability of widespread homologous recombination between *S*. *aureus* from different hosts may be high, and the potential of this agent to infect both humans and animals may indicate the chances of a greater or lesser degree of recombination or point mutation not having a noticeably clear pattern in *S*. *aureus*, unless characteristics other than the infection chain are intrinsically defined in the analysis metadata, such as those of ES-GPM.

The greater diversity and polymorphism of some alleles of *S*. *aureus* may be associated with adaptive mutations due to response to environmental changes or a switch in host species, since this agent presents tropism to several hosts and in particular, antibiotic exposure^50^. In addition, housekeeping genes are genes associated with metabolic maintenance, which demonstrates that adaptations in their genomes may be affecting metabolism in response to distinct nutrient availability^50^. Of all genes, *yqiL* and *aroE* showed the highest values of non-synonymous substitutions and mutations. The *yqiL* locus in *S*. *aureus* demonstrated a potential signature of recombination^45^ compared to the other six gene fragments^38^, but the role of this locus in infections is poorly understood. However, a high number of non-synonymous substitutions may suggest that the removal of deleterious mutations by purification selection should be relatively slow. Gene *aroE* has also been shown to contribute to the chronicity of *S*. *aureus* infection, such as the invasiveness and cytotoxicity of the agent, with an increase in the load of intracellular bacteria^51^, and because of that, this same locus can play an important role in the persistence of infection such as ES-GPM in goats. Moreover, the *aroE* gene locus is shown to be in a region of the genome with an excess of homologous recombination, most likely in MGEs^38^ and with that it can confer an increased capacity to colonize and infect ruminants^52^.

Interestingly, the *glpF* sequence, among all others, was shown to be more conserved, with the lowest haplotypic diversity and the highest average G + C content, and also presenting low numbers of mutations, even though it is present in a region of the genome with excess homologous recombination^38^. The highest average G + C content may be linked to the pathogenic potential for the host genome^53^. However, we can emphasize that the *glpF* gene has a role in the formation of the L form in bacteria^54^, which is directly involved in antibiotic tolerance or persistence^55^ in *S*. *aureus*, such as to ampicillin or norfloxacin^3^. This demonstrates that *glpF* sequence, even though it is a member of the metabolic housekeeping genes and is present in the core genome, it is directly linked to persistence in infections; therefore, a high *glpF* sequence polymorphism may not be advantageous for colonization of the host as in ES-GPM for enrofloxacin and thus is shown to be more conserved within the other six alleles.

## Conclusion

In this study we identified 27 specific genetic mutations for strains of ES-GPM (*S*. *aureus* isolated from goats with persistent mastitis) that may help in the future to discriminate *S*. *aureus* in cases of persistent goat mastitis (GPM). In addition, in 22 CCs that we found, CC133, CC5 and a new ST 4966 were specifically related to ES-GPM. We describe polymorphisms in specific alleles *arcC* and *glpF* genes, that showed a greatest number of mutations for ES-GPM in relation to non-ES-GPM. Furthermore, the identification of *S*. *aureus* and these polymorphisms genetics in persistent bacterial infections together with the MLST, can assist in decision making for the appropriate choice of protocol and of an antibiotic for the treatment of mastitis persists in goats. We hope that future studies can better clarify the persistence of this agent in certain antibiotic treatments.

## Methods

### Bacterial strains

In this study, we used two data sources, the first being cataloged by our research group with 18 isolates of *S*. *aureus* from goats diagnosed with GPM, recovered from milk samples, and treated with enrofloxacin antibiotic. The animals are kept under intensive farming in a free stall regime, with a high-level mechanical milking system and automatic cleaning of milk pipes. The harvest of samples was established in the Capril UFV, located in the mesoregion of Zona da Mata of Minas Gerais, Brazil. The isolates were identified by phenotypic and genotypic tests, as well as by methods of bacteriological examination and antibiotic sensitivity tests as previously reported^12,56^.

Briefly, the animals selected were examined and diagnosed with clinical mastitis caused by *S. aureus*. Firstly, were evaluated for signs of clinical mastitis and the presence of at least visually abnormal milk (i.e., the presence of flakes, clots, blood, or serous milk), as well as changes in the mammary gland, such as an increased volume and body temperature, and the presence of pain, redness during forestripping performed at the milking parlour, in the presence of a veterinarian. After antibiogram results, these animals were treated with enrofloxacin (KINETOMAX – Bayer). Minimal inhibitory concentrations (MICs) for enrofloxacin were performed using the Etest method (BioMérieux, Marcy l’Étoile, France). However, due to acute mastitis and the need for rapid treatment, only after completion of seven days of enrofloxacin treatment were these MIC results available. Twenty-one days after the completion of treatment, these animals continued to have clinical mastitis. New milk samples were collected, and *S. aureus* was isolated again. Therefore, the diagnosis of persistent mastitis (GPM) was based on the detection of the same species of agent in more than one consecutive sampling^57–59^. Thus, 18 isolates of *S. aureus* were obtained (nine before treatment and nine after treatment) and were grouped in this study as ES-GPM.

The second source of data for the analyzes were another 25 sequences of *S*. *aureus* isolates recovered from milk samples, these being selected from mastitis cases obtained from ovine and bovine isolates from the MLST online database (https://pubmlst.org/organisms/staphylococcus-aureus/). The search terms used were: country (Brazil), source (Milk) and disease (Mastitis), totaling 43 isolates from seven Brazilian states (Table S3).

### Genomic DNA extraction, MLST locus amplification and sequencing

The seven housekeeping genes of *S*. *aureus* isolates obtained from the GPMs were sequenced: carbamate kinase (*arcC*), shikimate dehydrogenase (*aroE*), glycerol kinase *(glpF)*, guanylate kinase (*gmk),* phosphate acetyltransferase *(pta),* triosephosphate isomerase *(tpi),* acetyl coenzyme A acetyltransferase (*yqiL*; Table S2), as previously described in methods and evaluated at https://pubmlst.org/organisms/staphylococcus-aureus/^60^. DNA from *S*. *aureus* isolates were extracted using the PROMEGA kit following the manufacturer's protocol and fragments amplified according to the protocol described by ENRIGHT et al.^61^. The gene and primer specifications are shown in Table S2. MLST locus amplification was performed in 50 µL reaction volumes containing 0.5 µL DNA, 0.5 µg of each primer, 1U Taq DNA polymerase (Qiagen, Crawley, UK), 5 µL buffer 103 (supplied with Taq Polymerase) and 0.2 mM deoxynucleoside triphosphates (Perkin-Elmer Applied Biosystems; Foster City, California). Initial denaturation was for 5 min at 95 °C, followed by 30 cycles at 55 °C for 1 min, extension at 72 °C for 1 min and denaturation at 95 °C for 1 min, followed by the final extension at 72 °C for 5 min. The amplified products were sent for sequencing at MACROGEN, INC. (Seoul, South Korea) using capillary electrophoresis.

### Alignment, editing and curation of *S*. *aureus* MLST sequences

The 18 forward and reverse sequences of the seven *S*. *aureus* MLST genes obtained from ES-GPM were trimmed and aligned with their respective allele type (AT) and sequence type (ST), obtained from the PubMLST database (https://pubmlst.org/organisms/staphylococcus-aureus/^21^ in the UGENE software^62^ along with the 25 deposited sequences.

### Expanded multi-locus sequence typing (E-MLST)

The ST, allelic profile and CC were confirmed by the E-MLST database (https://pubmlst.org/organisms/staphylococcus-aureus/)^21^. Any ST or allele not previously described were submitted to the database and assigned new AT and ST numbers.

### Setting the best-fit model of DNA evolution and phylogenetic tree

Phylogenetic reconstructions by Bayesian Inference (BI) and Maximum Likelihood (ML) require the setting of a DNA evolution model to calculate the probabilities of nucleotide changes^63^. “HKY + I” was selected as the best evolution model of the MLST genes by the jModeltest 2 program^64^. The phylogenetic tree was inferred by the Markov Chain Monte Carlo (MCMC) method using MrBayes v3.1.2^65^ and phylogenetic trees were calculated in two runs with 1,000,000 (one million) generations and a sampling frequency of 100 (one hundred). The parameter convergence was analyzed in Tracer version 1.6 (http://tree.bio.ed.ac.uk/software/tracer) and 25% of the trees generated were burned to produce the consensus tree. The phylogenetic tree and geospatial information was visualized together with associated metadata using Microreact Web server version 5.93.0^66^ and by Figtree software version 1.4.4^67^.

### Sequence diversity analyses

The G + C content, variable sites (Ss) or mutations (Ms), number of polymorphic sites, average pairwise nucleotide, number of singleton (SNPs) and difference per site (π) were calculated with DnaSP Version 6.12.03^68^. The average non-synonymous/synonymous rate ratio (dN/dS) was calculated with KaKs Calculator Version 2.0^69^ to infer the direction and magnitude of natural selection. The ratio of non-synonymous and synonymous substitutions (dN/dS) measures the level of selection in a protein-coding gene. Further, the dN/dS ratio indicates purifying selection if dN/dS < 1, positive selection if dN/dS > 1, and neutral evolution if values are close to 1^17^.

### Population structure and recombination analyses

Strain relationships were analyzed using the goeBURST algorithm^70^, as implemented in the software PHYLOViZ^71^ to cluster the STs into CCs based on the most stringent definition. Global optimal eBURST implemented by PHYLOViZ was used to cluster STs, generating a multi-locus sequence tree (MS tree) to visualize possible evolutionary relationships between STs. The pairwise homoplasy index (*phi*) test^72^ implemented in SplitTree4^73^ for recombination was performed, and a *P*-value of < 0.05 indicated that recombination existed.

The LDhat program^74^ implemented in Recombination Detection Program (RDP) v.4.97^75^ was used to calculate the per-site ρ/θ ratio based on the concatenated sequences of seven loci with 1,000,000 MCMC updates. The parameters ρ and θ represent the rates of recombination and mutation, respectively. Linkage disequilibrium from allelic data was evaluated by calculating the standardized index of association (IAs) using LIAN v3.735^76^ in web interface (http://guanine.evolbio.mpg.de/cgi-bin/lian/lian.cgi.pl/query). The null hypothesis of complete linkage equilibrium (IAs > 0; presence of linkage disequilibrium or clonality) was tested by using Monte Carlo methods with 10,000 iterations on allelic profile^17^. If there is linkage equilibrium because of frequent recombination events, the expected value of IAs is zero, which suggests no association between alleles at different loci; if IAs are statistically significant different from zero, alleles are suggested with genetic linkage^77^.

### Genetic network

Genetic networks present an alternative view of genealogies represented by bifurcated structures of phylogenetic trees, and the possible dispersal routes of *S*. *aureus* isolates in the Brazilian dairy milk were predicted following the methodology described by Vidigal et al.^78^. To reconstruct the network, sequences of AT and ST genes were grouped into haplotypes using DnaSP v6^68^. Following this, the network was constructed using the network 4.6.1.0 (http://www.fluxus-technology.com) and the Median Joining algorithm (MJ)^79^.

### Ethics statement

The experimental protocol was approved by the Ethics Committee (Comissão de ética no uso de animais - CEUA) of the Federal University of Viçosa, according to the protocol number 43/2016. The methods were carried out in accordance with the approved guidelines. In addition, this experiment was conducted by Bacterial Diseases Laboratory (LDBAC) and the Molecular Biology Laboratory (BIOMOL) located at the Veterinary Department (DVT) of the Universidade Federal de Viçosa (UFV), Viçosa, Minas Gerais.

## Supplementary Information

Supplementary Information.

## Data Availability

All data generated and/or analyzed during this study are included in this published article, and at Microreact interactive viewer^66^ [https://microreact.org/project/puA-MDz8o]. Additionally, the accession numbers of the sequences or reference codes used in this study are called IDs and can be seen in the supplementary tables S1 and S3, with their isolated characteristics and their corresponding AT and ST. These data are publicly available and accessible online at the *S*. *aureus* PubMLST database [https://pubmlst.org/organisms/staphylococcus-aureus] (IDs: 33768 - 33785)^21^.
