# Supplementary material for: Multilocus sequence analysis reveals genetic diversity in Staphylococcus aureus isolate of goat with mastitis persistent after treatment with enrofloxacin
Source: Sci Rep. 2021 Aug 26;11:17252. doi: 10.1038/s41598-021-96764-z (PMC8390490; doi:10.1038/s41598-021-96764-z)
Supplement: Supplementary file 1 — Supplementary Information. [file 41598_2021_96764_MOESM1_ESM.docx]

**Multilocus sequence analysis reveals genetic diversity in *Staphylococcus aureus* isolate of goat with mastitis persistent after treatment with enrofloxacin**

Richard Costa Polveiro^1^, Manuela Maria Cavalcante Granja^1^, Thais Coimbra Borba Roldão^1^, Ilderlane Da Silva Lopes^1^, Pedro Marcus Pereira Vidigal^2^, Magna Coroa Lima^1^, Maria Aparecida Scatamburlo Moreira^1*^.

^1^ Laboratory of Bacterial Diseases, Sector of Preventive Veterinary Medicine and Public Health, Veterinary Department, Universidade Federal de Viçosa, Viçosa, Minas Gerais 336570-900 Brazil.

^2^ Núcleo de Análise de Biomoléculas (NuBioMol), Center of Biological Sciences, Universidade Federal de Viçosa, Viçosa, Minas Gerais 336570-900 Brazil.

* E-mail: [masm@ufv.br](mailto:masm@ufv.br)

**Supplementary Fig. S**
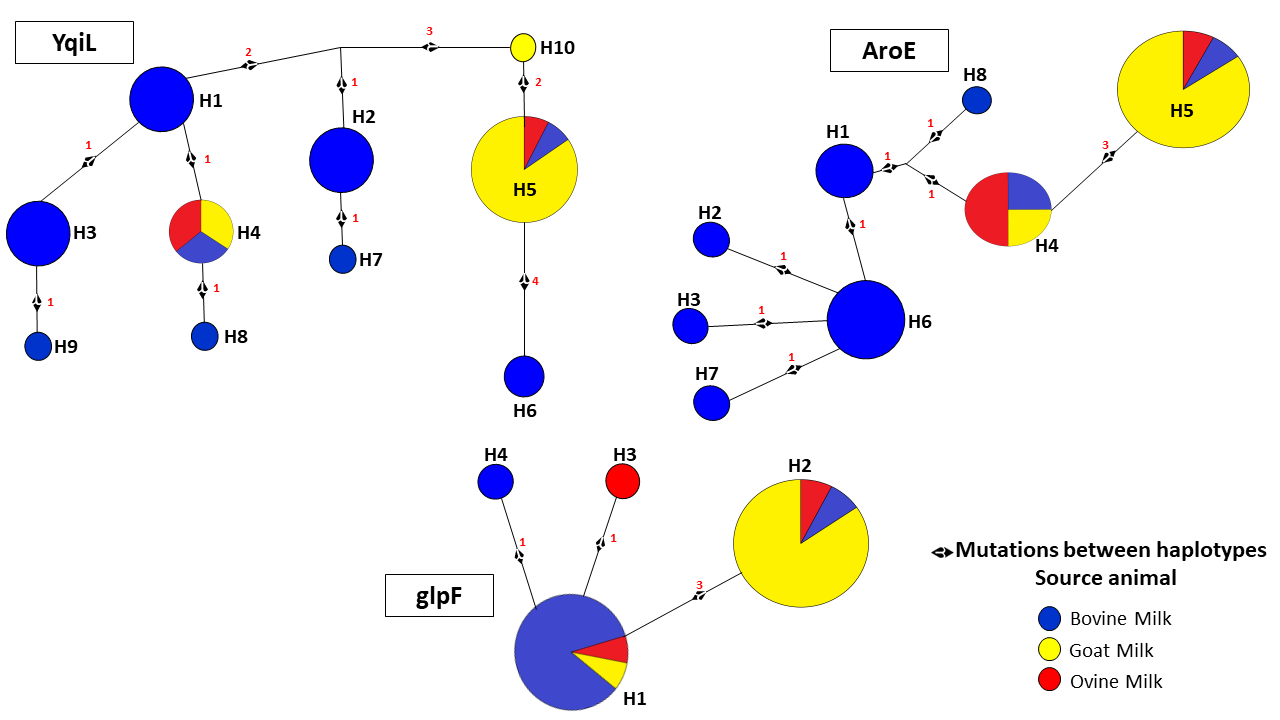
**1** Haplotypic network of *yqiL*, *aroE* and *glpF* of multilocus sequence typing of *Staphylococcus aureus* obtained from milk of animals with mastitis in Brazil.

Red numbers between haplotypes - mutations sites.

**yqiL**

**yqiL**

**Supplementary Fig. S2** Haplotypic network of *arcC*, *tpi*, *pta* and *gmK* of *Staphylococcus aureus* multilocus sequence typing obtained from milk of animals with mastitis in Brazil.


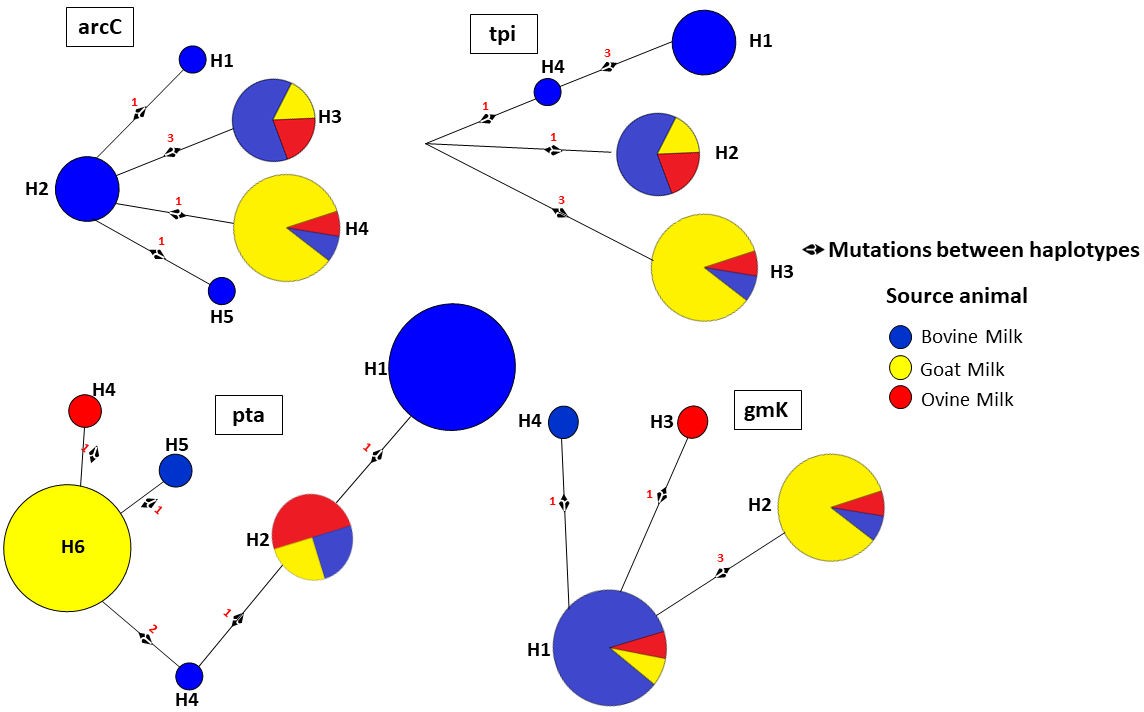


Red numbers between haplotypes - mutations sites

**Supplementary Table S1.** Identification of the 43 isolates of *Staphylococcus aureus* used in the MLST.

| **ID** | **State** | **Year** | **Disease** | **Source** | **ST** |
| --- | --- | --- | --- | --- | --- |
| 1551 | Rio de Janeiro | 2002 | Mastitis | Bovine milk | 741 |
| 1552 | Rio de Janeiro | 2001 | Mastitis | Bovine milk | 742 |
| 1553 | Rio de Janeiro | 2001 | Mastitis | Bovine milk | 743 |
| 1554 | Rio de Janeiro | 2001 | Mastitis | Bovine milk | 744 |
| 1555 | Rio de Janeiro | NA | Mastitis | Bovine milk | 745 |
| 1556 | Rio de Janeiro | 2002 | Mastitis | Bovine milk | 746 |
| 1557 | Rio de Janeiro | 2004 | Mastitis | Bovine milk | 747 |
| 3445 | Pernambuco | 2007 | Mastitis | Ovine milk | 1728 |
| 3446 | Pernambuco | 2007 | Mastitis | Ovine milk | 1729 |
| 3447 | Pernambuco | 2007 | Mastitis | Ovine milk | 1730 |
| 4809 | São Paulo | 2010 | Mastitis | Bovine milk | 2493 |
| 5518 | Pernambuco | 2004 | Mastitis | Bovine milk | 2987 |
| 5519 | Pernambuco | 2004 | Mastitis | Bovine milk | 2988 |
| 6019 | Pernambuco | 2004 | Mastitis | Bovine milk | 3257 |
| 6020 | Pernambuco | 2004 | Mastitis | Bovine milk | 3258 |
| 31643 | Minas Gerais | 2010 | Mastitis | Bovine milk | 126 |
| 31644 | Rio Grande do Sul | 2010 | Mastitis | Bovine milk | 746 |
| 31645 | Paraná | 2010 | Mastitis | Bovine milk | 1 |
| 31646 | Paraná | 2010 | Mastitis | Bovine milk | 1 |
| 31647 | Paraná | 2010 | Mastitis | Bovine milk | 1 |
| 31648 | Santa Catarina | 2010 | Mastitis | Bovine milk | 746 |
| 31649 | Santa Catarina | 2010 | Mastitis | Bovine milk | 188 |
| 31650 | Santa Catarina | 2010 | Mastitis | Bovine milk | 8 |
| 31651 | São Paulo | 2010 | Mastitis | Bovine milk | 126 |
| 31652 | São Paulo | 2010 | Mastitis | Bovine milk | 126 |
| 33768 | Minas Gerais-LDBAC | 2014 | Mastitis - GPM | Goat milk | 133 |
| 33769 | Minas Gerais- LDBAC | 2014 | Mastitis - GPM | Goat milk | 133 |
| 33770 | Minas Gerais- LDBAC | 2014 | Mastitis - GPM | Goat milk | 5 |
| 33771 | Minas Gerais- LDBAC | 2014 | Mastitis - GPM | Goat milk | 133 |
| 33772 | Minas Gerais- LDBAC | 2014 | Mastitis - GPM | Goat milk | 133 |
| 33773 | Minas Gerais- LDBAC | 2014 | Mastitis - GPM | Goat milk | 133 |
| 33774 | Minas Gerais- LDBAC | 2014 | Mastitis - GPM | Goat milk | 133 |
| 33775 | Minas Gerais- LDBAC | 2014 | Mastitis - GPM | Goat milk | 133 |
| 33776 | Minas Gerais- LDBAC | 2014 | Mastitis - GPM | Goat milk | 133 |
| 33777 | Minas Gerais- LDBAC | 2014 | Mastitis - GPM | Goat milk | 133 |
| 33778 | Minas Gerais- LDBAC | 2014 | Mastitis - GPM | Goat milk | 133 |
| 33779 | Minas Gerais- LDBAC | 2014 | Mastitis - GPM | Goat milk | 133 |
| 33780 | Minas Gerais- LDBAC | 2014 | Mastitis - GPM | Goat milk | 133 |
| 33781 | Minas Gerais- LDBAC | 2014 | Mastitis - GPM | Goat milk | 133 |
| 33782 | Minas Gerais- LDBAC | 2014 | Mastitis - GPM | Goat milk | 133 |
| 33783 | Minas Gerais- LDBAC | 2014 | Mastitis - GPM | Goat milk | 133 |
| 33784 | Minas Gerais- LDBAC | 2014 | Mastitis - GPM | Goat milk | 4966 |
| 33785 | Minas Gerais- LDBAC | 2014 | Mastitis - GPM | Goat milk | 133 |

List of isolates used in the study, subdivided by identification in database (ID), states of Brazil, year of sample collection, disease or type of mastitis, source of sample and sequence type (ST).

**Supplementary Table S2.** Table of genes fragments and primer specifications used in sequencing.

| Gene | *primer* | Sequence (5′-3′) |
| --- | --- | --- |
| Carbamate kinase (arcC) | arcC-Up  arcC-Dn | TTGATTCACCAGCGCGTATTGTC  AGGTATCTGCTTCAATCAGCG |
| Shikimate dehydrogenase (aroE) | aroE-Up  aroE-Dn | ATCGGAAATCCTATTTCACATTC  GGTGTTGTATTAATAACGATATC |
| Glycerol kinase (glpF) | glpF-Up  glpF-Dn | CTAGGAACTGCAATCTTAATCC  TGGTAAAATCGCATGTCCAATTC |
| Guanylate kinase (gmk) | gmk-Up  gmk-Dn | ATCGTTTTATCGGGACCATC  TCATTAACTACAACGTAATCGTA |
| Phosphate acetyltransferase (pta) | pta-Up  pta-Dn | GTTAAAATCGTATTACCTGAAGG  GACCCTTTTGTTGAAAAGCTTAA |
| Triosephosphate isomerase (tpi) | tpi-Up | TCGTTCATTCTGAACGTCGTGAA |
| Acetyl coenzyme A acetyltransferase (yqiL) | yqiL-Up  yqiL-Dn | CAGCATACAGGACACCTATTGGC  CGTTGAGGAATCGATACTGGAAC |

Genes and oligonucleotides used to detect the seven housekeeping genes of *S*. *aureus* isolated from caprine mastitis.

Source: ENRIGHT *et al.*, 2000

Supplementary Table S3. Table of characterization by MLST of isolates identified as *Staphylococcus aureus* persistent for enrofloxacina and other isolates of different types of mastitis used in this study.

| ID | State | Year | Source | Disease | *arcC* | *aroE* | *glp* | *gmk* | *pta* | *tpi* | *yqiL* | ST |
| --- | --- | --- | --- | --- | --- | --- | --- | --- | --- | --- | --- | --- |
| 1551 | Rio de Janeiro | 2002 | Bovine milk | Mastitis | 88 | 68 | 1 | 4 | 1 | 5 | 40 | 741 |
| 1552 | Rio de Janeiro | 2001 | Bovine milk | Mastitis | 3 | 131 | 1 | 1 | 1 | 5 | 3 | 742 |
| 1553 | Rio de Janeiro | 2001 | Bovine milk | Mastitis | 1 | 130 | 1 | 1 | 1 | 1 | 1 | 743 |
| 1554 | Rio de Janeiro | 2001 | Bovine milk | Mastitis | 1 | 4 | 1 | 72 | 12 | 1 | 10 | 744 |
| 1555 | Rio de Janeiro | NA | Bovine milk | Mastitis | 6 | 66 | 46 | 2 | 99 | 50 | 18 | 745 |
| 1556 | Rio de Janeiro | 2002 | Bovine milk | Mastitis | 3 | 1 | 1 | 1 | 1 | 5 | 92 | 746 |
| 1557 | Rio de Janeiro | 2004 | Bovine milk | Mastitis | 3 | 1 | 1 | 1 | 1 | 5 | 96 | 747 |
| 3445 | Pernambuco | 2007 | Ovine Milk | Mastitis | 1 | 4 | 220 | 4 | 12 | 1 | 10 | 1728 |
| 3446 | Pernambuco | 2007 | Ovine Milk | Mastitis | 6 | 66 | 46 | 2 | 182 | 50 | 18 | 1729 |
| 3447 | Pernambuco | 2007 | Ovine Milk | Mastitis | 1 | 4 | 1 | 4 | 12 | 1 | 180 | 1730 |
| 4809 | São Paulo | 2010 | Bovine milk | Mastitis | 1 | 1 | 1 | 1 | 1 | 1 | 284 | 2493 |
| 5518 | Pernambuco | 2004 | Bovine milk | Mastitis | 3 | 68 | 1 | 4 | 1 | 5 | 3 | 2987 |
| 5519 | Pernambuco | 2004 | Bovine milk | Mastitis | 330 | 68 | 1 | 4 | 1 | 5 | 40 | 2988 |
| 6019 | Pernambuco | 2004 | Bovine milk | Mastitis | 3 | 1 | 429 | 1 | 1 | 5 | 3 | 3257 |
| 6020 | Pernambuco | 2004 | Bovine milk | Mastitis | 3 | 468 | 1 | 1 | 1 | 5 | 3 | 3258 |
| 31643 | Minas Gerais | 2010 | Bovine milk | Mastitis | 3 | 68 | 1 | 4 | 1 | 5 | 40 | 126 |
| 31644 | Rio Grande do Sul | 2010 | Bovine milk | Mastitis | 3 | 1 | 1 | 1 | 1 | 5 | 92 | 746 |
| 31645 | Paraná | 2010 | Bovine milk | Mastitis | 1 | 1 | 1 | 1 | 1 | 1 | 1 | 1 |
| 31646 | Paraná | 2010 | Bovine milk | Mastitis | 1 | 1 | 1 | 1 | 1 | 1 | 1 | 1 |
| 31647 | Paraná | 2010 | Bovine milk | Mastitis | 1 | 1 | 1 | 1 | 1 | 1 | 1 | 1 |
| 31648 | Santa Catarina | 2010 | Bovine milk | Mastitis | 3 | 1 | 1 | 1 | 1 | 5 | 92 | 746 |
| 31649 | Santa Catarina | 2010 | Bovine milk | Mastitis | 3 | 1 | 1 | 8 | 1 | 1 | 1 | 188 |
| 31650 | Santa Catarina | 2010 | Bovine milk | Mastitis | 3 | 3 | 1 | 1 | 4 | 4 | 3 | 8 |
| 31651 | São Paulo | 2010 | Bovine milk | Mastitis | 3 | 68 | 1 | 4 | 1 | 5 | 40 | 126 |
| 31652 | São Paulo | 2010 | Bovine milk | Mastitis | 3 | 68 | 1 | 4 | 1 | 5 | 40 | 126 |
| 33768 | Minas Gerais-LDBAC | 2014 | Goat milk | Mastitis - GPM | 6 | 66 | 46 | 2 | 7 | 50 | 18 | 133 |
| 33769 | Minas Gerais-LDBAC | 2014 | Goat milk | Mastitis - GPM | 6 | 66 | 46 | 2 | 7 | 50 | 18 | 133 |
| 33770 | Minas Gerais-LDBAC | 2014 | Goat milk | Mastitis - GPM | 6 | 66 | 46 | 2 | 7 | 50 | 18 | 5 |
| 33771 | Minas Gerais-LDBAC | 2014 | Goat milk | Mastitis - GPM | 6 | 66 | 46 | 2 | 7 | 50 | 18 | 133 |
| 33772 | Minas Gerais-LDBAC | 2014 | Goat milk | Mastitis - GPM | 6 | 66 | 46 | 2 | 7 | 50 | 18 | 133 |
| 33773 | Minas Gerais-LDBAC | 2014 | Goat milk | Mastitis - GPM | 6 | 66 | 46 | 2 | 7 | 50 | 18 | 133 |
| 33774 | Minas Gerais-LDBAC | 2014 | Goat milk | Mastitis - GPM | 6 | 66 | 46 | 2 | 7 | 50 | 18 | 133 |
| 33775 | Minas Gerais-LDBAC | 2014 | Goat milk | Mastitis - GPM | 6 | 66 | 46 | 2 | 7 | 50 | 18 | 133 |
| 33776 | Minas Gerais-LDBAC | 2014 | Goat milk | Mastitis - GPM | 6 | 66 | 46 | 2 | 7 | 50 | 18 | 133 |
| 33777 | Minas Gerais-LDBAC | 2014 | Goat milk | Mastitis - GPM | 6 | 66 | 46 | 2 | 7 | 50 | 18 | 133 |
| 33778 | Minas Gerais-LDBAC | 2014 | Goat milk | Mastitis - GPM | 6 | 66 | 46 | 2 | 7 | 50 | 18 | 133 |
| 33779 | Minas Gerais-LDBAC | 2014 | Goat milk | Mastitis - GPM | 6 | 66 | 46 | 2 | 7 | 50 | 18 | 133 |
| 33780 | Minas Gerais-LDBAC | 2014 | Goat milk | Mastitis - GPM | 6 | 66 | 46 | 2 | 7 | 50 | 18 | 133 |
| 33781 | Minas Gerais-LDBAC | 2014 | Goat milk | Mastitis - GPM | 6 | 66 | 46 | 2 | 7 | 50 | 18 | 133 |
| 33782 | Minas Gerais-LDBAC | 2014 | Goat milk | Mastitis - GPM | 6 | 66 | 46 | 2 | 7 | 50 | 18 | 133 |
| 33783 | Minas Gerais-LDBAC | 2014 | Goat milk | Mastitis - GPM | 6 | 66 | 46 | 2 | 7 | 50 | 18 | 133 |
| 33784 | Minas Gerais-LDBAC | 2014 | Goat milk | Mastitis - GPM | 6 | 66 | 46 | 2 | 7 | 50 | 5 | 4966 |

Characterization for isolates in state of origin, year, disease, alleles type (AT) and sequence types (ST).

Supplementary Table S4 Nucleotide and allelic sequence diversity of *S*. *aureus* persistent for enrofloxacina

| **Locus** | **Length (bp)** | **Average G + C content (%)** | **S/M** | **No. of polymorphic sites (%)** | **S/M compared to non-MPC** | **Nucleotide diversity (π)** |
| --- | --- | --- | --- | --- | --- | --- |
| ***arcC*** | 456 | 38.20% | 4 | 0.88% | 66% | 0.00097 |
| ***aroE*** | 443 | 30.30% | 8 | 1.81% | 57% | 0.00195 |
| ***glpF*** | 465 | 41.10% | 3 | 0.65% | 60% | 0.00072 |
| ***gmk*** | 417 | 33.33% | 4 | 0.96% | 33% | 0.00107 |
| ***pta*** | 474 | 36.30% | 3 | 0.63% | 50% | 0.00070 |
| ***tpi*** | 402 | 37.30% | 4 | 0.99% | 57% | 0.00111 |
| ***yqiL*** | 516 | 37.60% | 8 | 1.55% | 54% | 0.00210 |

Variable sites (**S**)/mutations(**M**); Nucleotide diversity, Pi (**π**); contains 18 alleles of MPC.
